# Object-of-Interest Perception in a Reconfigurable Rolling-Crawling Robot

**DOI:** 10.3390/s22145214

**Published:** 2022-07-12

**Authors:** Archana Semwal, Melvin Ming Jun Lee, Daniela Sanchez, Sui Leng Teo, Bo Wang, Rajesh Elara Mohan

**Affiliations:** 1 Engineering Product Development, Singapore University of Technology and Design, Singapore 487372, Singapore; archana_semwal@sutd.edu.sg (A.S.); melvin_lee@sutd.edu.sg (M.M.J.L.); daniela_sanchez@mymail.sutd.edu.sg (D.S.); 22yteos695a@student.ri.edu.sg (S.L.T.); rajeshelara@sutd.edu.sg (R.E.M.); 2 Information Systems Technology and Design, Singapore University of Technology and Design, Singapore 487372, Singapore

**Keywords:** shape reconfigurable robots, locomotion mode, environment perception, object-of-interest, deep learning, computer vision

## Abstract

Cebrenus Rechenburgi, a member of the huntsman spider family have inspired researchers to adopt different locomotion modes in reconfigurable robotic development. Object-of-interest perception is crucial for such a robot to provide fundamental information on the traversed pathways and guide its locomotion mode transformation. Therefore, we present a object-of-interest perception in a reconfigurable rolling-crawling robot and identifying appropriate locomotion modes. We demonstrate it in Scorpio, our in-house developed robot with two locomotion modes: rolling and crawling. We train the locomotion mode recognition framework, named Pyramid Scene Parsing Network (PSPNet), with a self-collected dataset composed of two categories paths, unobstructed paths (e.g., floor) for rolling and obstructed paths (e.g., with person, railing, stairs, static objects and wall) for crawling, respectively. The efficiency of the proposed framework has been validated with evaluation metrics in offline and real-time field trial tests. The experiment results show that the trained model can achieve an mIOU score of 72.28 and 70.63 in offline and online testing, respectively for both environments. The proposed framework’s performance is compared with semantic framework (HRNet and Deeplabv3) where the proposed framework outperforms in terms of mIOU and speed. Furthermore, the experimental results has revealed that the robot’s maneuverability is stable, and the proposed framework can successfully determine the appropriate locomotion modes with enhanced accuracy during complex pathways.

## 1. Introduction

Living beings possess the ability to coordinate and adapt their gaits to walk on various complicated pathways. Various creatures use multiple locomotion modes and switch to appropriate locomotion types to overcome various pathway challenges. Inspiration from such creatures has tremendously enhanced mechanism design and overcoming traditional limitations in field robotics. Further, a dynamically changing environment has increased locomotion challenges for the robots to operate effectively. Thus, robots need to perceive objects of interests in the pathways and features in the surrounding, recognize the context of the situation and plan their locomotion mode, path, and interaction accordingly. Finally, determining the appropriate locomotion mode will improve the synergy of the robot and the surrounding environments.

This paper presents a semantic segmentation-based approach to perceive object-of-interest of indoor environment and determine the appropriate rolling or crawling morphology locomotion modes of our in-house developed shape-shifting robot ‘Scorpio’. To the best of our knowledge, there is no direct framework to recognize appropriate locomotion modes using visual features and a convolutional neural network.

This paper is organized as follows; Section 1 and Section 2 present the introduction and literature review. Section 3 provides the methodology and the overview of the proposed system. The experimental setup, findings, and discussion are covered in Section 4 and Section 5. Finally, Section 6 concludes this research work.

## 2. Related Work

In literature, various reconfigurable robots have been reported to dynamically adapt to environment changing. Reconfigurable robots have potential applications such as search and rescue, de-mining, environmental monitoring, and planetary exploration. Here, the application of bio-inspired principles leads to adaptive, flexible interaction and improves the performance limitations of fixed dimension robots. In [1], a quadruped wheeled robot Tarantula with variable wheel footprint kinematics was introduced by Aamir et al. for the specific geometry of the drain. In another study, Aamir et al. [2] designed a reconfigurable pavement sweeping robot Panthera for different pavement width conditions. In [3], Ilyas et al. presented a novel, modular, and reconfigurable staircase cleaning robot named sTetra. In [4], Vega et al. designed a modular window facade cleaning robot called Mantis. In [5], Jayaram and Full introduced a cockroach exoskeleton-inspired robot to explore confined environments. In [6], Peyer et al. presented bio-inspired magnetic swimming microrobots for biomedical applications. An inchworm-inspired crawling robot controlled by shape memory alloy was introduced by Shi et al. [7]. Further, in [8], Lin et al. introduced a caterpillar-inspired soft-bodied rolling robot named GoQBot. In [9], the authors presented an integrated jumping-crawling robot using a height-adjustable jumping module. In [10], a Salamander-inspired robot was presented that can change its locomotion mode from swimming to walking depending on the terrain it is traversing. The literature survey shows that reconfigurability can help overcome obstacles or continue task performance and showcase the structural capabilities despite changing pathways and environments. However, object-of-interest perception and transitions between locomotion modes remain a significant challenge faced by a reconfigurable robot.

In the past decade, environment sensing techniques like surface electromyography (EMG), radar detectors, laser rangefinders, and inertial measurement units (IMUs) have been used to develop automated locomotion mode recognition system [11,12]. However, some of these techniques can be inconvenient and may have bias errors. Recently, researchers have also used Decision Trees (DT) [13,14], Support Vector Machines (SVM) [15,16,17], Neural Networks (NN) [18,19,20] for solving environment perception problems. Among these techniques, NNs based frameworks are the most popular and widely used by many researchers in many different applications. Further, NNs have many types such as Multi-layer Feed-forward Neural Network (MLFFNN), Recurrent Neural Network (RNN), Radial Basis Function (RBF), General Regression Neural Network (GRNN), Probabilistic Neural network (PNN), Complementary Neural Network (CMTNN), and Space Invariant Artificial Neural Networks (SIANN) or Convolutional Neural Network (CNN) [21]. Here, CNNs has features like parameter sharing and dimensionality reduction which reduces the computational power needed. Thus, CNNS have the potential to solve object-of-interest perception or scene recognition tasks. In [22], Suryamurthy et al. proposed path planning framework for wheeled-legged robot CENTAURO. The authors employed a single RGB-based deep neural network to predict pixel-wise terrain labels and help reconfiguration for safe traversal among obstacles. In [2], Yi et al. presented a vision-based reconfiguration of a self-reconfigurable pavement sweeping robot called Panthera, which can adjust its frame width to ease the cleaning tasks to become friendly with different pavement geometry. In [23], Aslan et al. developed a deep learning algorithm for humanoid robots to walk to the target using semantic segmentation and a deep Q network. In [24], Doan et al. proposed a semantic segmentation network with residual depth-wise separable blocks to detect street objects such as cars and pedestrians. In [25], Kowalewski et al. presented the object-level semantic perception of the environment for indoor mobile robots. The experiments results indicated that the proposed framework, the Mask-RCNN, achieved the mAP score of 0.414. In [26], Bersan et al. proposed a semantic segmentation-based approach to localize and identify different classes of the objects in the scene. In [27], Dvornik et al. presented a deep real-time network named BlitzNet for scene understanding. In [28], Deng et al. presented a vision-based navigation method for a small-scale quadruped robot named Pegasus-Mini. The authors trained ERFNet framework with cityscape, garden, and cityscape-garden datasets and confirmed satisfactory results. In [29], Belter et al. proposed a motion planning framework for a robot by employing natural terrain semantics. The above mentioned studies mainly use semantic segmentation based framework as it clusters the parts of the images together which belong to the same class. Further, this technique can be exploited for object-of-interest perception. However, most research in reconfigurable robots was limited to mechanism design and control studies with minimal effort related to switching locomotion modes. Object-of-interest is a key characteristic essential for the most reconfigurable robots that allows for recognizing appropriate locomotion modes. Therefore, this study presents object-of-interest perception to determine appropriate locomotion mode in a reconfigurable rolling-crawling robot.

## 3. Overview of the Proposed System

**Figure 1:**
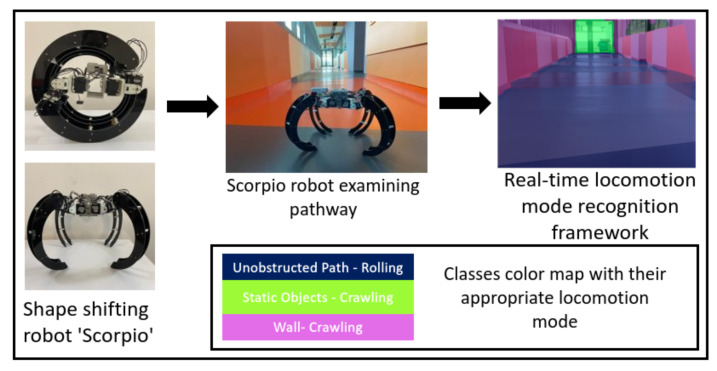
Overview diagram of proposed framework.

Figure 1 presents the overview of the locomotion mode recognition framework. Here, a semantic segmentation-based approach was used to determine the appropriate locomotion mode of the Scorpio shape-shifting robot. As shown in Figure 1, Scorpio robot has two locomotion modes: rolling during unobstructed pathways and crawling during obstructed pathways. The robot perceives object-of-interest of indoor using real-time locomotion mode recognition framework. This framework segments the environment into different classes of unobstructed pathways and obstructed pathway. As a result, the robot is able to choose appropriate locomotion mode with respect to the pathway being traversed. The details of robot architecture and locomotion mode recognition framework are described below.

### 3.1. Semantic Segmentation Framework

**Figure 2:**
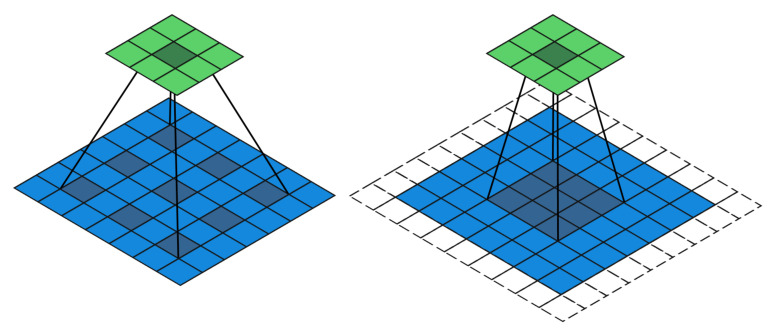
Dilated convolution (**left**) and conventional convolution (**right**).

Pyramid Scene Parsing Network (PSPNet) [30] contains two parts: an encoder and a decoder. The encoder is responsible for extracting the features uses a ResNet101 backbone with dilated convolutions and a pyramid pooling module. The features extracted from the ResNet101 backbone is downsampled 8 times before dilated convolutions and pyramid pooling are applied. The last two stages of the ResNet101 backbone replace the traditional convolutional layers with dilated convolution layers where the dilation factor K equals 2 and 4, respectively. Compared to conventional CNN kernel, dilated convolution injects zeros into it’s kernel to help increase the receptive field resulting in richer features. Figure 2 shows an example of a 3×3 dilated convolution that has K equivalent to 2 against conventional convolution.

**Figure 3:**
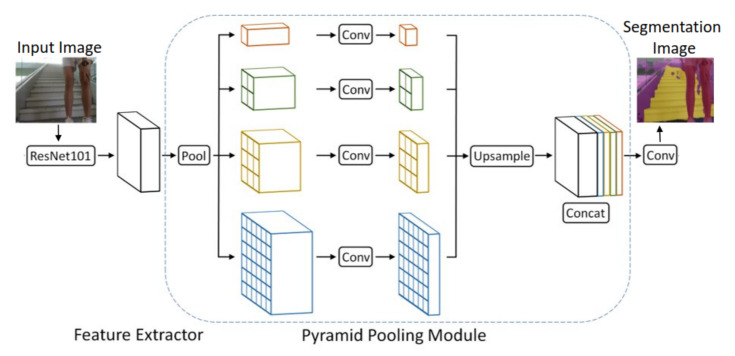
Pyramid pooling module.

The pyramid pooling module helps the model capture a more global context of images. This is done by pooling the feature map from the ResNet101 backbone with different sizes. Upsampling ensures the output is the same size as the original feature map. The original feature map is then concatenated with the different sized upsampled pooling feature maps. Figure 3 shows the pyramid pooling module. The decoder then takes the features and converts them into predictions. The decoder used after the pyramid pooling module is an 8 times bilinear upsampling decoder as the features were initially downsampled by 8 times.

### 3.2. Physical Layer

The main scope of inspiration for the Scorpio robot is a Cebrennus Rechenburgi spider capable of switching rolling and crawling locomotion modes [31]. Further, the Scorpio robot is defined as a quadruped robot with a spider’s appearance and is divided into two regions, namely the body and limbs. The body consists of the control and power units with four limbs attached. Each limb has three active joints powered by servo motors, and these active joints consolidate a multi-joint structure that gives each limb three Degrees of Freedom (DOF). This ensures the necessary motions for the reconfigurable platform, such as crawling and rolling locomotion modes. Finally, the distal servo motor is attached to a double-layered 5 mm acrylic leg. All the specifications of the Scorpio robot are detailed in Table 1. The details of the two locomotion modes are as follows.

#### 3.2.1. Locomotion Module

**Figure 4:**
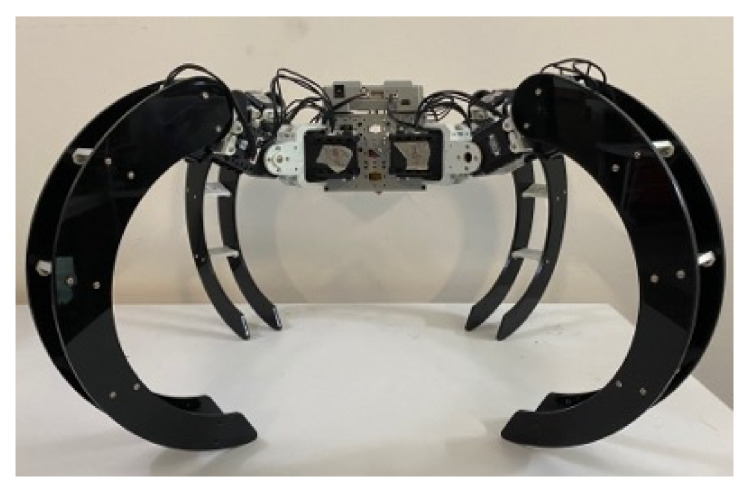
Crawling configuration.

The Scorpio robot has two modes of locomotion, crawling and rolling; the rotation limits constrain these locomotion modes from each servo motor. When the robot is crawling, each servo motor of the multi-joint structure delivers rotational motion that, in combination, generates forward, clockwise, counter-clockwise and reverse movements of the legs. The motions and the legs’ positions allow the robot to move forward and make left-right turns. Figure 4 shows the Scorpio robot in crawling locomotion mode.

**Figure 5:**
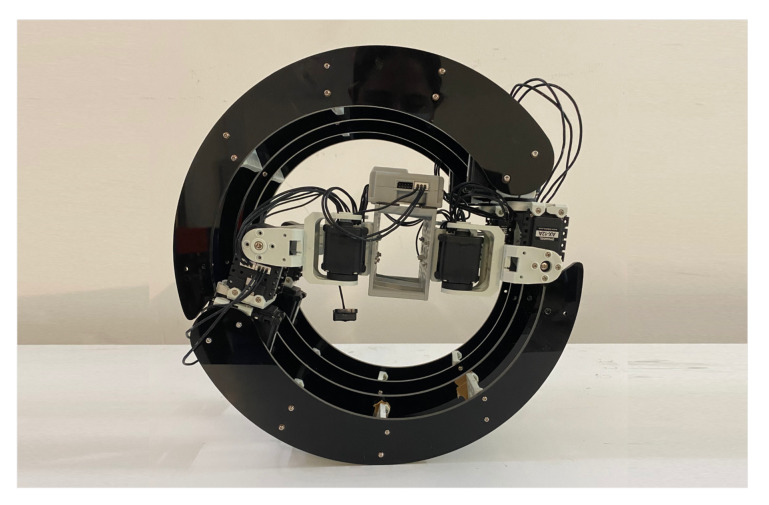
Rolling configuration.

In the rolling locomotion, the frontal legs move to the center while the multi-joint structure allows them to go below the body, and the back legs move to the back center and involve the body from the top, creating a shell. This configuration results in a circular shape, and the robot moves the two legs that are in contact with the surface to generate the rolling movement. The mechanism of this module is divided into two halves. The first half of the servo motor engages in direct contact with the ground while and the second half engages in touch with the ground after the first half’s revolution. The Figure 5 shows the configuration of the rolling locomotion.

#### 3.2.2. System Architecture

**Figure 6:**
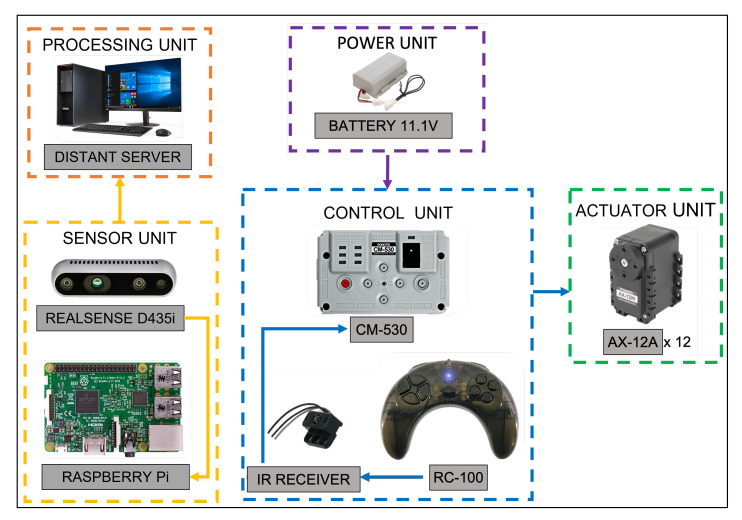
System architecture.

In this version of the Scorpio robot platform, the BIOLOID system was implemented, including the Robo+ software for programming the interactions between the components of the system and the configuration of the servomotors for each step on the robot’s gait. As shown in the system architecture diagram in Figure 6, the control unit used is the CM-530, powered by an 11.1 V 900 mA LiPo battery. Using a remote controller and an IR receptor, we made the twelve AX-12A DYNAMIXEL smart actuators move to different positions, allowing the robot to change between crawling and rolling locomotion modes; and a Realsense camera connected to a Raspberry Pi to send the captured images via WIFI to a distant server for processing the collected data.

## 4. Experimental Setup & Results

This section describes the experimental setup and results of the proposed framework. The experiments were carried out in five phases: dataset preparation and training of the proposed framework, validation of the Scorpio’s performance in the indoor environment, evaluating the trained model on both offline and real-time field tests, comparing the trained model with other semantic frameworks and validating the proposed framework in false ceiling environment.

### 4.1. Data-Set Preparation and Training

**Figure 7:**
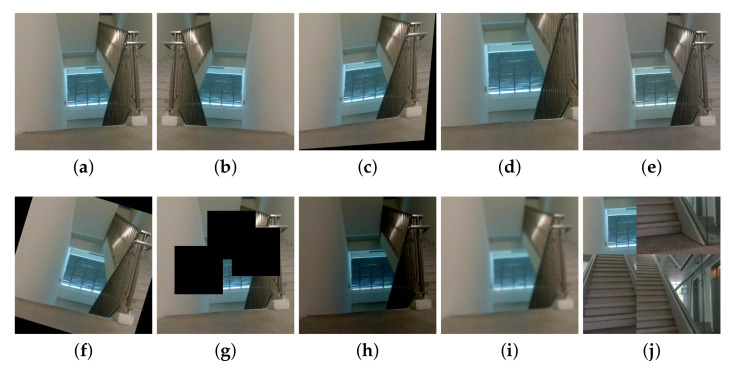
Data-augmentation example. (**a**) Original image. (**b**) Flip. (**c**) Shear. (**d**) Scale. (**e**) Contrast. (**f**) Rotation. (**g**) Cut out. (**h**) Brightness. (**i**) Blur. (**j**) Mosaic.

The training dataset of the Scorpio semantic segmentation framework for indoor environment is categorized into two groups: unobstructed paths (i.e., floor) and obstructed paths (e.g., with persons, railing, stairs, static objects, walls). Here, an unobstructed path is ideal for rolling locomotion mode, and the obstructed pathway is adequate for crawling locomotion mode. For indoor environment, the dataset consists of 100 images of each categories, resulting in a total of 453 training images where multiple classes co-exist in a single image in some cases. The dataset consists purely of the real-time collected dataset from the perspective of Scorpio within the Singapore University of Technology and Design campus. The images were resized to 512×512 pixel resolution before augmentation. First, the semantic segmentation ground truth was labeled using CVAT [32]. Then, data augmentation is applied to the training dataset to control over-fitting, resulting in 4530 images. Data augmentation processes such as scaling, rotation, horizontal flip, color enhancement, blurring, brightness, shearing, cutout, and mosaic are applied. Figure 7 shows the sample of data augmentation of one image. Table 2 elaborates the settings of the various types of augmentation applied.

#### 4.1.1. Training Hardware and Software Details

The Scorpio semantic segmentation algorithm is trained using the PyTorch library. PSPNet was pre-trained on the ImageNet dataset consisting of 1000 classes using the ResNet101 architecture. Stochastic Gradient Descent (SGD) optimizer was used to train the model. The hyper-parameters used are 0.9 for momentum, an initial learning rate of 0.01, and weight decay at 0.0005. Here, the learning rate of 0.01 was selected through the use of a learning rate range test starting from a very small learning rate 0.00001 all the way to a large learning rate 0.1. Similarly for momentum, short runs of training were done with 0.99, 0.97, 0.95 and 0.9. Once the learning rate and momentum were fixed, weight decay was tested from 0, 0.005, 0.001, 0.0005, 0.0001, 0.00005 and 0.00001. The model is trained for a total epoch of 2300 using a batch size of 4 before early stopping and validating the model in real-time inference. The model was trained and tested on the Lenovo ThinkStation P510. It consists of an Intel Xeon E5-1630V4 CPU running at 3.7 GHz, 64 GB Random Access Memory (RAM), and Nvidia Quadro P4000 GPU (1792 Nvidia CUDA Cores with 8 GB GDDR5 memory size running at 192.3 GBps bandwidth).

#### 4.1.2. Evaluation Metrics

The efficiency of our trained model was evaluated in offline and real-time test scenarios. Standard metrics were used to evaluate the semantic segmentation performance. Pixel accuracy and intersection over union (IoU) as defined in Equation (1) and (2), respectively were used to evaluate the model. In the equations, true positive, true negative, false positive and false negative are *tp*, *tn*, *fp* and *fn*, respectively.

(1) $$\mathrm{Pixel}\;\mathrm{Accuracy}\;\left(\mathrm{Acc}\right)=\frac{tp+tn}{tp+fp+tn+fn}$$

(2) $$\mathrm{Intersection}\;\mathrm{over}\;\mathrm{union}\;\left(\mathrm{IoU}\right)=\frac{tp}{tp+fp+fn}$$

### 4.2. Offline Test

**Figure 8:**
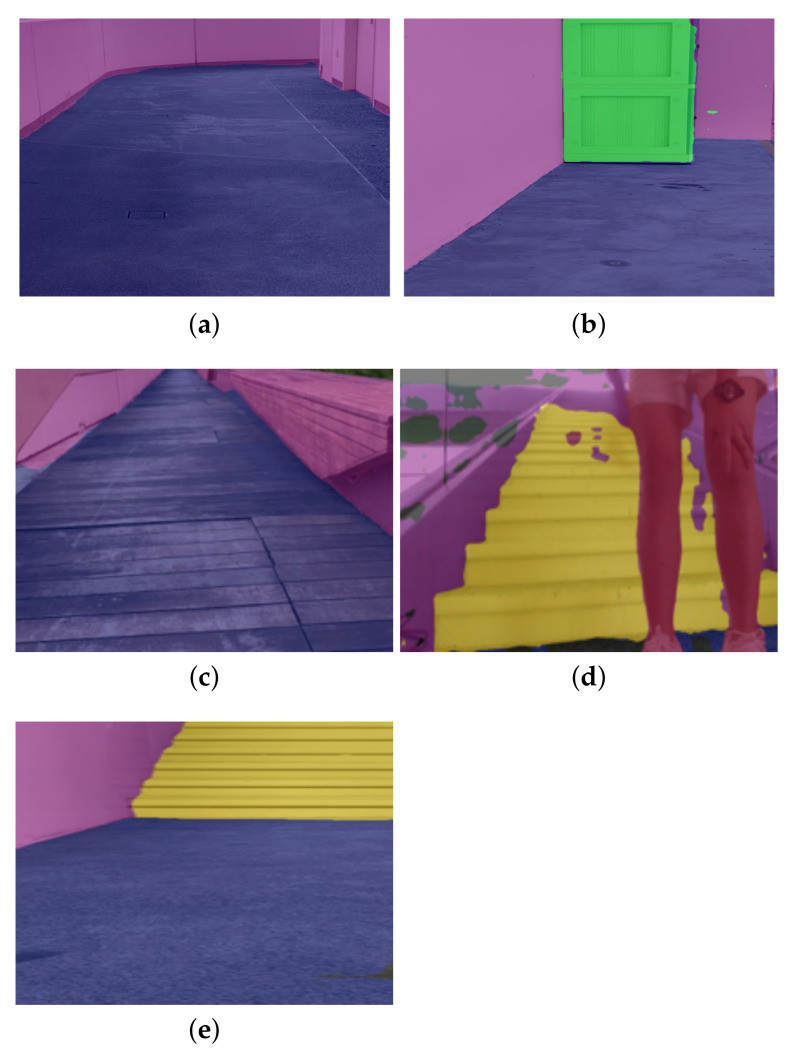
The offline test results of the locomotion mode recognition framework. (**a**) Floor and wall. (**b**) Floor, wall, and static object. (**c**) Floor and wall. (**d**) Stairs, person, and wall. (**e**) Floor, stair, and wall.

The offline test was performed to determine the efficiency of the locomotion mode recognition framework using the test dataset. In this evaluation process, 50 images were tested from the test dataset. Figure 8 show the results of the locomotion mode recognition framework. Here, the classes of the locomotion mode recognition framework such as the floor, person, railing, stairs, static objects (door, table, chair), and walls are denoted by the blue, red, orange, yellow, green, and purple, respectively. As mentioned earlier, the class floor is considered as an ideal unobstructed path for rolling locomotion mode. Table 3 demonstrates the results of the offline test.

It was observed that the framework identifies the appropriate locomotion mode with an average pixel accuracy of 87.35. Classes floor, person, railing, stairs, static objects, and walls were classified with an IoU score of 86.2, 89.6, 64.5, 71.3, 62.8 and 59.3, respectively. The proposed framework achieved an mIoU (mean IoU) of 72.28. The miss detection, false classification, and detection with lower pixel accuracy happened only for partially occluded objects. Hence, the framework is accurate for identifying appropriate locomotion modes for dynamically changing pathways.

### 4.3. Real-Time Field Trial

The real-time field trial experiments include validating Scorpio’s performance and evaluating the locomotion mode recognition framework using Scorpio-collected real-time pathway video feed.

#### 4.3.1. Validation of Scorpio’s Performance

**Figure 9:**
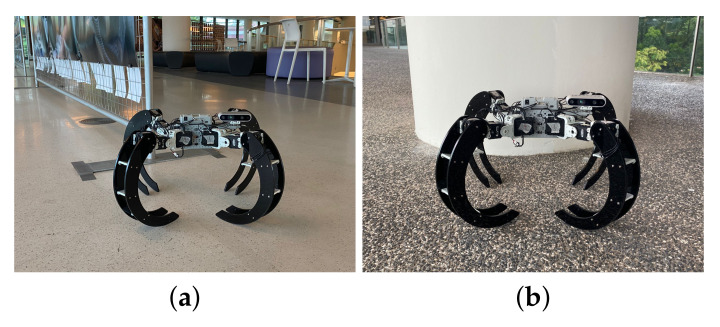
Crawling locomotion mode. (**a**) On smooth surface. (**b**) On rough surface.

**Figure 10:**
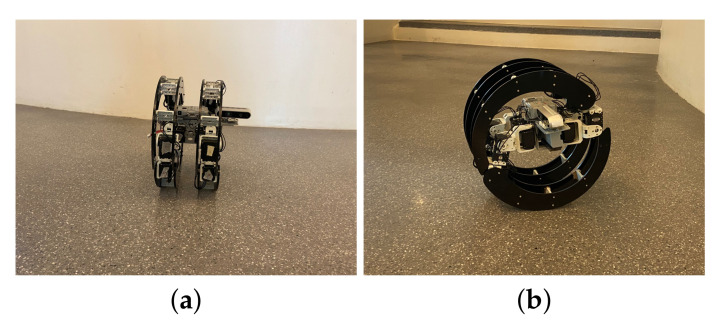
Rolling locomotion mode. (**a**) Front view. (**b**) Side view.

**Figure 11:**
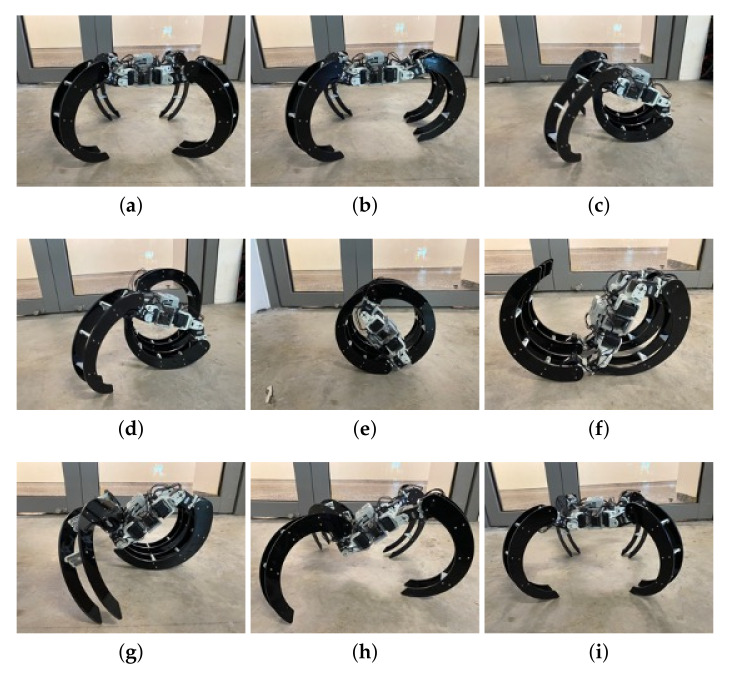
Transformation from crawling to rolling and back to crawling mode. (**a**) Crawling mode. (**b**) Frontal legs side by side. (**c**) Frontal legs under robot’s body. (**d**) Left back leg over robot’s body. (**e**) Rolling mode (frontal legs under robot’s body and back legs over it). (**f**) Back legs pushed out. (**g**) Back legs in crawling position. (**h**) Frontal legs pushed out and separated. (**i**) Crawling mode.

In this section, the robot’s performance was validated by evaluating the two locomotion modes, including rolling and crawling. During the experiment, the robot autonomously wandered around a given space in explore mode. The crawling locomotion of the Scorpio robot was tested on flat and rough terrain (Figure 9). Similarly, we performed the rolling locomotion of Scorpio in a teleoperated mode (Figure 10). In the entire course of our experiment, the Scorpio robot effectively exhibited both crawling and walking modes of locomotion. In addition, the experiments also verified the recovery and transformation gaits that allow switching between locomotion modes. Figure 11 shows the step-by-step transformation of the robot’s crawling to rolling and back to crawling. Further, it can move around the complex pathway and accurately capture images for the proposed real-time locomotion recognition.

#### 4.3.2. Real-Time Locomotion Mode Recognition Framework

**Figure 12:**
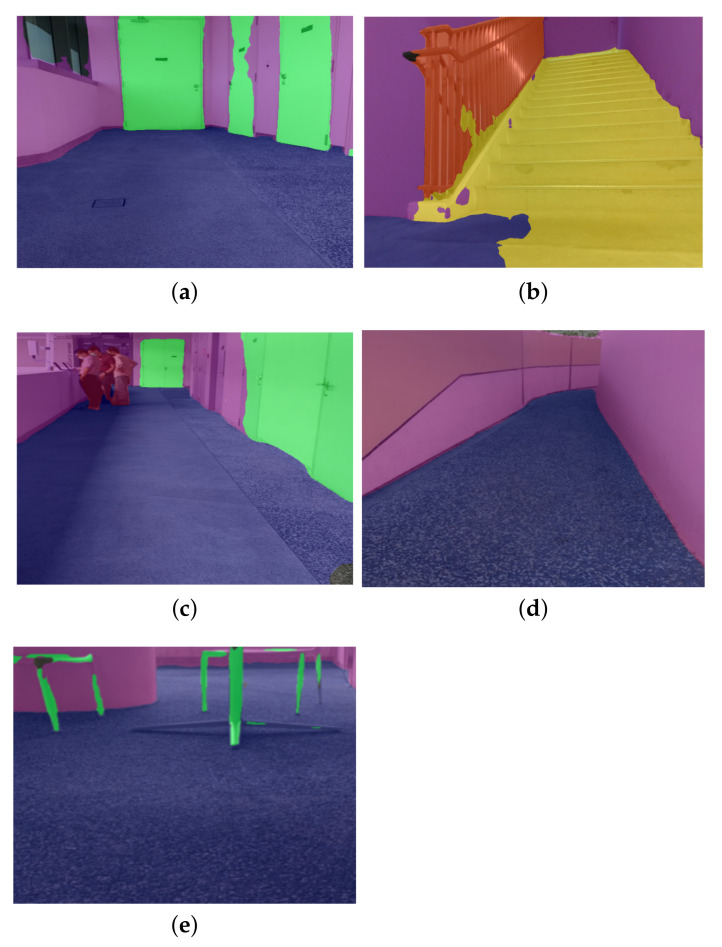
The online test results of the locomotion mode recognition framework. (**a**) Floor, static object, and wall. (**b**) Railing, stairs, floor and wall. (**c**) Floor, person, static object, and wall. (**d**) Floor and wall. (**e**) Floor, static object, and wall.

This section evaluates the locomotion mode recognition framework in the real-time field trial. The experiments were carried out in an unexplored environment on the SUTD campus. Here, an unexplored environment has not been used for data-set collection. In our experiments, the Scorpio robot captured the pathway images using its onboard camera after every rolling and crawling cycle to capture better quality images. The captured images are transmitted over WiFi to a high-powered GPU-enabled local server for recognizing appropriate locomotion modes. Figure 12 depicts the test results of the locomotion mode recognition framework. Table 4 shows the statistical measure results of the locomotion mode recognition framework.

The experimental results demonstrate that the locomotion mode recognition framework classified obstructed and unobstructed paths with an average pixel accuracy of 86.5. The proposed framework’s performance is also accurate with respect to object boundaries. The evaluation metric indicates that the framework has detected classes floor, person, railing, stairs, static object, and walls with an mIoU score of 70.63. Miss classification is attributed to blurring caused by the jerks when traversing on uneven ground and other factors.

## 5. Comparison and Validation

It includes the performance comparison of proposed algorithm with other semantic frameworks and existing works. Further, it validates the performance of the locomotion mode recognition framework of the Scorpio robot to inspect false-ceiling environment.

### 5.1. Comparison with Other Semantic Frameworks

**Figure 13:**
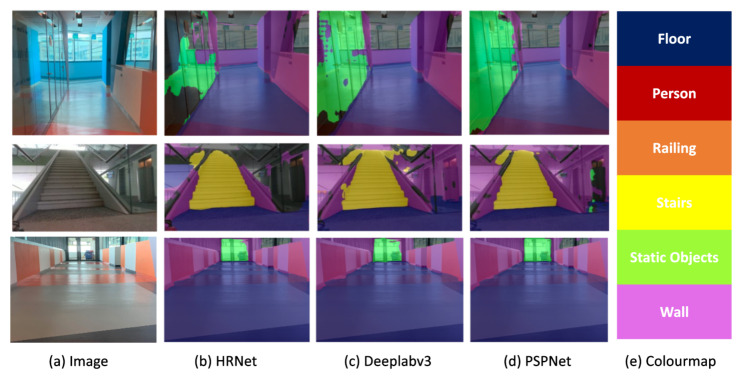
Comparison with other semantic frameworks.

This section illustrates the performance comparison of the proposed locomotion mode recognition framework with other popular semantic segmentation frameworks. Here, HRNet [33] and Deeplabv3 [34] are considered for semantic segmentation model comparison analysis. Figure 13 shows the experimental results of the three models. Table 5 summarizes the details of comparison analysis. The comparison analysis is based on the common evaluation metrics for semantic image segmentation. The results of the evaluation metrics demonstrate that the proposed framework outperformed with an mIOU score of 72.28 and a speed of 96.59 ms. The experimental analysis indicates that HRNet and Deeplabv3 have comparatively less mIOU and PSPNet yields better pixel-wise classification than other networks. The multi-branch parallel structure of HRNet can effectively gather spatial information, but it ignores global context, and boundary information [35]. The Deeplabv3 model’s performance was lower due to poor segmentation results along object boundaries. Whereas the PSPNet architecture considers the global context of the image to generate the local level predictions, resulting in better performance. Moreover, few errors in PSPNet are completely related to the illusion created by the reflection of railing glasses.

### 5.2. Comparison with Other Existing Works

This section elaborates the comparative analysis of the proposed algorithm with other existing scene classification studies reported in the literature. To our best knowledge, there is no direct study to perceive object-of-interest and determine the appropriate locomotion mode based on robotic vision. Further, Table 6 states the accuracy of various scene classifications based on some similar classes.

The literature has reported various studies focusing on scene classification. However, the implementations in these case studies cannot be directly compared to our work. The case studies have employed different training datasets, CNN algorithms, training parameters, and performed offline inspection. Further, the accuracy of our proposed framework is comparatively same, and the proposed framework has a key feature of performing real-time locomotion mode recognition.

### 5.3. Validation in False-Ceiling Environment

False-ceiling inspection has become essential to ensure the commercial building and human safety. Typically, a false ceiling is built with material like Gypsum board, Plaster of Paris, Poly Vinyl Chloride (PVC), and used to hide ducting, messy wires, and Heating, Ventilation, and Air Conditioning (HVAC) system. The poor construction of false ceiling environment can lead to early deterioration and unexplained odours. Human visual inspection of false ceiling environment faces lots of challenges due to requirement of a highly-skilled labour, safety issue and workforce shortage. These facts highlight the need for an automated inspection of false ceiling environment. Hence, the aim of this section is to train and validate the performance of locomotion mode recognition framework of the Scorpio robot to inspect false-ceiling environment.

**Figure 14:**
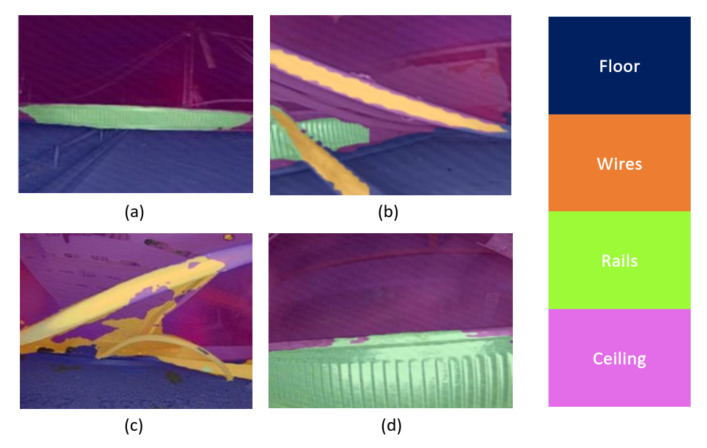
Offline test results of the locomotion mode recognition framework’s in the false ceiling environment: (**a**) Recognition of floor, rails and ceiling, (**b**) Recognition of floor, ceiling, rails and wire, (**c**) Recognition of floor, ceiling and wires, and (**d**) Recognition of floor and rails.

In this experimental section, the proposed framework was trained and tested for the false ceiling environment. Here, the training dataset was composed of two categories: unobstructed path (i.e., floor) and obstructed path (e.g., ceiling, rails, and wires). The dataset was self-collected from the perspective of Scorpio and consist of 100 images of each categories. The images were resized to 512 × 512 pixel resolution and fed into the data augmentation algorithm to control over-fitting issue. The training hardware and software details were the same as explained in Section 4.1.1. Further, in the evaluation process, 50 images were tested from the test dataset. Figure 14 shows the results of locomotion mode recognition framework in the false ceiling environment. Here the classes floor, ceiling, rails, and wires were denoted by blue, purple, green, and yellow, respectively. Table 7 provide the statistical results.

It was observed that the framework identifies the appropriate locomotion mode in the false ceiling environment with an average pixel accuracy of 85.52%. Classes floor, rails, walls, and wires were classified with an IoU score of 83.2, 79.3, 60.1, 55.3, respectively. This framework has achieved an mIoU score of 67.36. Hence, the framework is accurate for identifying appropriate locomotion mode in complex false ceiling environment. Moreover, the Scorpio robot with locomotion mode recognition framework can easily traverse obstacles and help false ceiling inspection tasks.

## 6. Conclusions

A semantic segmentation-based approach was presented for recognizing the appropriate locomotion modes in the shape-reconfigurable robot Scorpio. The experimental setup has included validating Scorpio’s performance and evaluating the locomotion mode recognition framework using Scorpio-collected real-time pathway video feed. The robot’s maneuverability is stable on smooth and rough terrain. Furthermore, the locomotion recognition framework was tested on test dataset and real-time pathway images collected by the Scorpio robot. The experimental results show that PSPNet is able to detect obstructed paths and unobstructed paths with a mIoU score of 72.28, 12.6% and 3.4% higher than the scores of HRNet and Deeplabv3, respectively. It takes only 96.59 ms to process one image on the local server that is the fastest among the counterparts. Therefore, the proposed method is more advantageous to recognize the appropriate locomotion modes for shape reconfigurable robots. Further, in this study, there is a big emphasis on understanding and predicting the object-of-interest to further improve the synergy of robot and surrounding environment. The capability to autonomously determine appropriate locomotion mode is critical for meeting goals around quality, cost, efficiency and speed. In our future work, we plan to further improve the performance of the locomotion mode recognition framework with event-based sensors due to exponential growth in the demand for automated solutions.

## Figures and Tables

**Table 1:** Technical specifications of Scorpio.

Description	Specification
Dimension (Crawling)	46 cm × 46 cm × 27 cm
Dimension (Rolling)	29.5 cm diameter
Weight (including battery)	1.3 kg
Full Body Material	Acrylic
Smart Actuators	Dynamixel AX-12A (12 no’s)
Working Voltage	7.4 V
Maximum Obstacle Height	0.3 cm
Operational Duration	45 min
Battery	11.1 V
Camera	Realsense D435i

**Table 2:** Augmentation type and setting.

Augmentation Type	Augmentation Setting
Scaling	0.5× to 1.5×
Rotation	from −45 degree to +45 degree
Horizontal flip	flip the image horizontally
Color enhancing	contrast (from 0.5× to 1.5×)
Blurring	Gaussian Blur (from sigma 1.0× to 3.0×)
Brightness	from 0.5× to 1.5×
Shear	*x* axis (−30 to 30) *y* aixs (−30 to 30)
Cutout	1 to 3 squares up to 35% of pixel size
Mosaic	random crop and combination of 4 images

**Table 3:** Statistical measures for offline locomotion mode recognition.

Category	Class	Pixel Accuracy	IoU	mIoU
Unobstructed Path (Rolling)	Floor	92.5	86.2	
	Person	93.4	89.6	
	Railing	82.9	64.5	72.28
Obstructed Path (Crawling)	Stairs	88.6	71.3	
	Static object	83.6	62.8	
	Walls	83.1	59.3	

**Table 4:** Statistical measures for online locomotion mode recognition.

Category	Class	Pixel Accuracy (%)	IoU	mIOU
Unobstructed Path (Rolling)	Floor	91.9	84.6	
	Person	92.5	87.6	
	Railing	82.2	62.6	70.63
Obstructed Path (Crawling)	Stairs	87.8	70.1	
	Static object	82.9	61.1	
	Walls	81.8	57.8	

**Table 5:** Comparison with other semantic segmentation framework.

Semantic Framework	Pixel Accuracy (%)	mIOU	Speed (ms)
PSPNet (Proposed framework)	87.35	72.28	96.59
HRNet	78.1	64.17	158.59
Deeplabv3	84.5	69.89	98.53

**Table 6:** Comparison with other existing works.

Case Studies	Classification Type	Algorithm	Classes	mIOU
Rafique et al. [36]	Offline	Linear SVM	11	72.2
Lopez et al. [37]	Offline	Two-branched CNN and Attention Module	61	74.04
Couprie et al. [38]	Offline	Multiscale Convolutional Network	14	52.4
Proposed framework	Real-time with Scorpio	PSPNet	6	70.63

**Table 7:** Statistical measures for locomotion mode recognition framework in the false ceiling environment.

Category	Class	Pixel Accuracy (%)	IoU	mIOU
Unobstructed Path (Rolling)	Floor	89.2	83.2	
	Rails	88.5	79.3	67.36
	Walls	81.1	60.1	
Obstructed Path (Crawling)	Wires	85.2	55.3	

## Data Availability

We would like to share the data only on users request.
